# A pore is a pore is a pore (or a hub?): VDAC oligomerization in mitochondrial connectivity and modulation

**DOI:** 10.1042/BST20250480

**Published:** 2026-06-03

**Authors:** Vito De Pinto, Giuseppe Battiato, Stefano Conti-Nibali, Salvatore Antonio Maria Cubisino

**Affiliations:** Department of Biomedical and Biotechnological Sciences, University of Catania, Catania, Italy

**Keywords:** bioenergetics, mitochondria, molecular interactions, molecular ultrastructure, voltage-gated channels

## Abstract

For decades, the voltage-dependent anion-selective channel (VDAC), formerly known as the mitochondrial porin, was considered a simple pore enabling nearly free permeability across the outer mitochondrial membrane. This simplified view has been progressively dismantled through the discovery of three mammalian isoforms (VDAC1, VDAC2, and VDAC3) with the gradual attribution, often serendipitous, of diverse cellular roles beyond passive metabolite exchange. Recent advances in cryo-electron microscopy have catalyzed a breakthrough in VDAC research. Three converging lines of evidence are reshaping our understanding: (a) high-resolution structures of VDAC within its native protein complexes; (b) discovery of unexpected functions, including phospholipid scrambling and regulation of outer membrane permeabilization through higher-order oligomeric assemblies; and (c) structural determination of VDAC interactions with macromolecules, as well as small-molecule modulators. Collectively, these insights have strengthened the consideration of VDAC as a multifunctional signaling hub and therapeutic target, with emerging small molecules and peptides designed to modulate gating, oligomerization, and interfering with interacting partners. The aim of this review is to summarize current structural, functional, and pharmacological advances in VDAC biology, emphasizing how oligomerization dynamics and isoform specificity orchestrate mitochondrial behavior and offering perspectives on therapeutic strategies for diseases driven by mitochondrial dysfunction.

## Introduction

Mitochondria are central hubs of cellular metabolism, signaling, and fate determination, integrating bioenergetic demands with stress responses and apoptotic pathways. At the interface between the cytosol and the mitochondrial intermembrane space resides the voltage-dependent anion-selective channel (VDAC), the most abundant protein of the outer mitochondrial membrane (OMM) and the principal conduit for metabolites, nucleotides, and ions. Originally characterized as a passive pore facilitating ATP/ADP exchange, VDAC is now recognized as a multifunctional platform coordinating mitochondrial homeostasis, inter-organelle communication, programmed cell death, and inflammation.

In mammals, three distinct VDAC isoforms have been identified as VDAC1, VDAC2, and VDAC3. Although these isoforms share a high degree of structural homology, they differ in their primary amino acid sequences, which may give rise to distinct functional properties and interaction profiles [1–3]. VDAC1, the first isoform to be resolved by X-ray crystallography and NMR spectroscopy, adopts a β-barrel architecture composed of 19 antiparallel β-strands, except for the first and nineteenth, which are parallel [4–6]. The N-terminal region, encompassing the first 26 residues, forms a characteristic α-helix directly connected to the first β-strand. The remaining β-strands are interconnected by short loop regions composed of small amino acid sequences. Structural information of VDAC2 is largely derived from X-ray crystallography of the zebrafish ortholog [7], even though the fish isoform resembles the mammalian VDAC1 isoform more, lacking the 11-amino acid N-terminal extension and showing only one cysteine residue. Human VDAC2 structure was recently determined by cryo-electron microscopy (cryo-EM) together with other interacting partners [8]. No experimental structural data are currently available for VDAC3. A growing body of structural and functional investigations shows higher-order oligomeric VDAC complexes. Although the arrangement and interfaces involved vary among the reports available so far, there are growing functional assignments to oligomeric VDAC in mitochondrial stress, in membrane permeability regulation, and even in the release of apoptogenic factors and mitochondrial DNA (mtDNA). Furthermore, phospholipid scrambling across the OMM has been recently attributed to dimers of VDAC. VDAC activity is further fine-tuned by numerous endogenous ligands, linking channel function to metabolic remodeling, inter-organelle associations, and stress signaling in an isoform-dependent manner. Beyond physiological regulation, VDACs have emerged as compelling pharmacological targets through newly identified small molecules and peptides which modulate channel gating, oligomerization, and protein–protein interactions.

This review synthesizes recent structural, mechanistic, and pharmacological insights into VDAC function, with a particular focus on oligomerization dynamics, isoform-specific roles, and emerging strategies for therapeutic targeting. Oligomerization has now emerged from speculation and modeling to be given a precise definition. This development is reminiscent of the moment in 2008 when the true structure of VDAC1 was published, which differed from the structures of bacterial porins and other hypotheses.

## Structural insights of VDAC isoforms and their oligomerization arrangement

**Figure 1:**
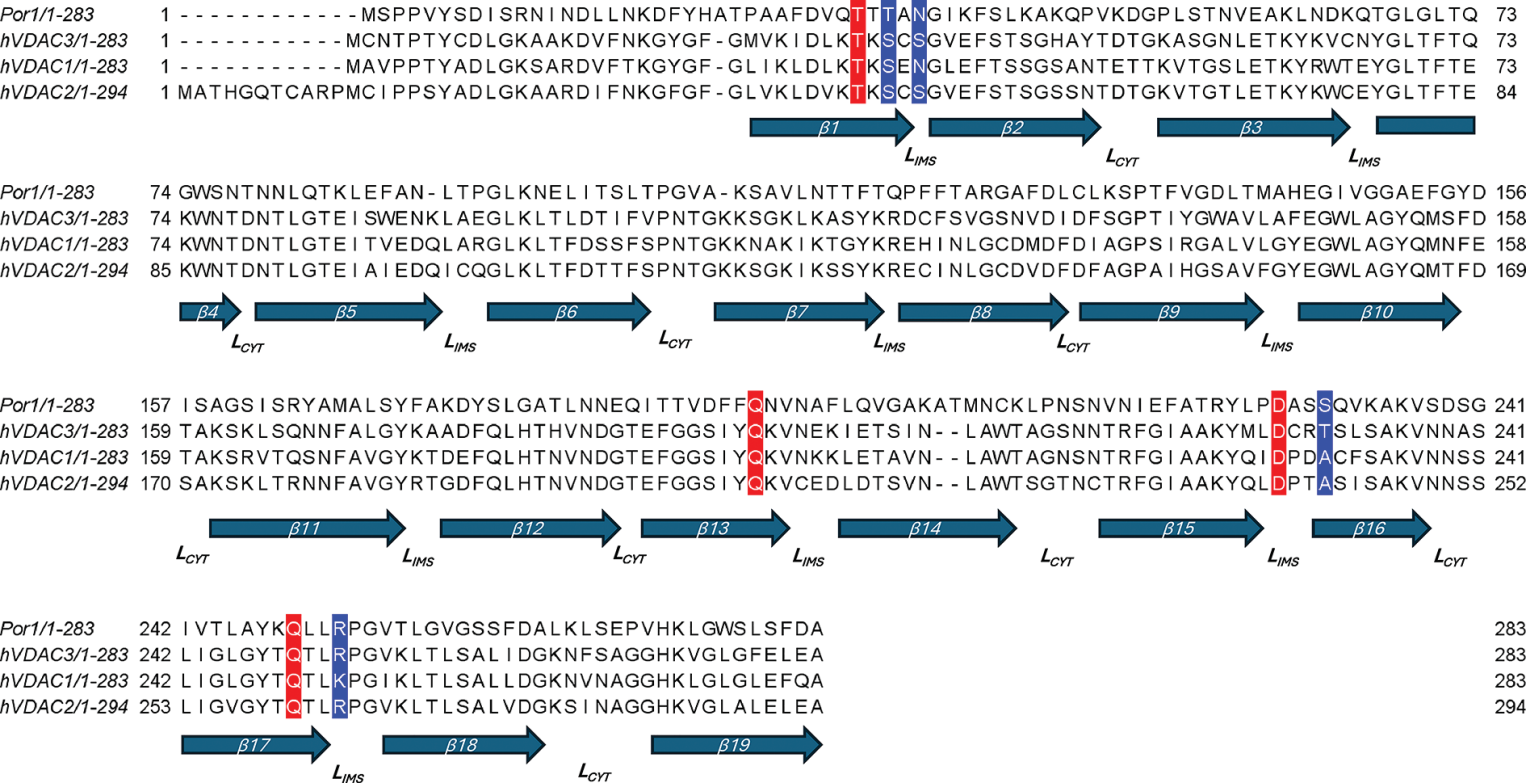
Multiple sequence alignment (MSA) of yeast Por1 and human VDAC isoforms (VDAC1, VDAC2, and VDAC3) The alignment highlights the conservation of residues involved in the formation of the hexameric oligomer structure. Additionally, the localization of each residue within the β-strands (indicated by blue arrows) is shown, alongside the exposure of each loop toward hydrophilic moiety (downward arrow: intermembrane space; upward arrow: cytosolic space). The MSA was performed using the ClustalW algorithm. Accession number used in the alignment: VDAC1: NP_001387937; VDAC2: NP_001171752; VDAC3: NP_001400481; Por1: NP_014343.1.

**Figure 2:**
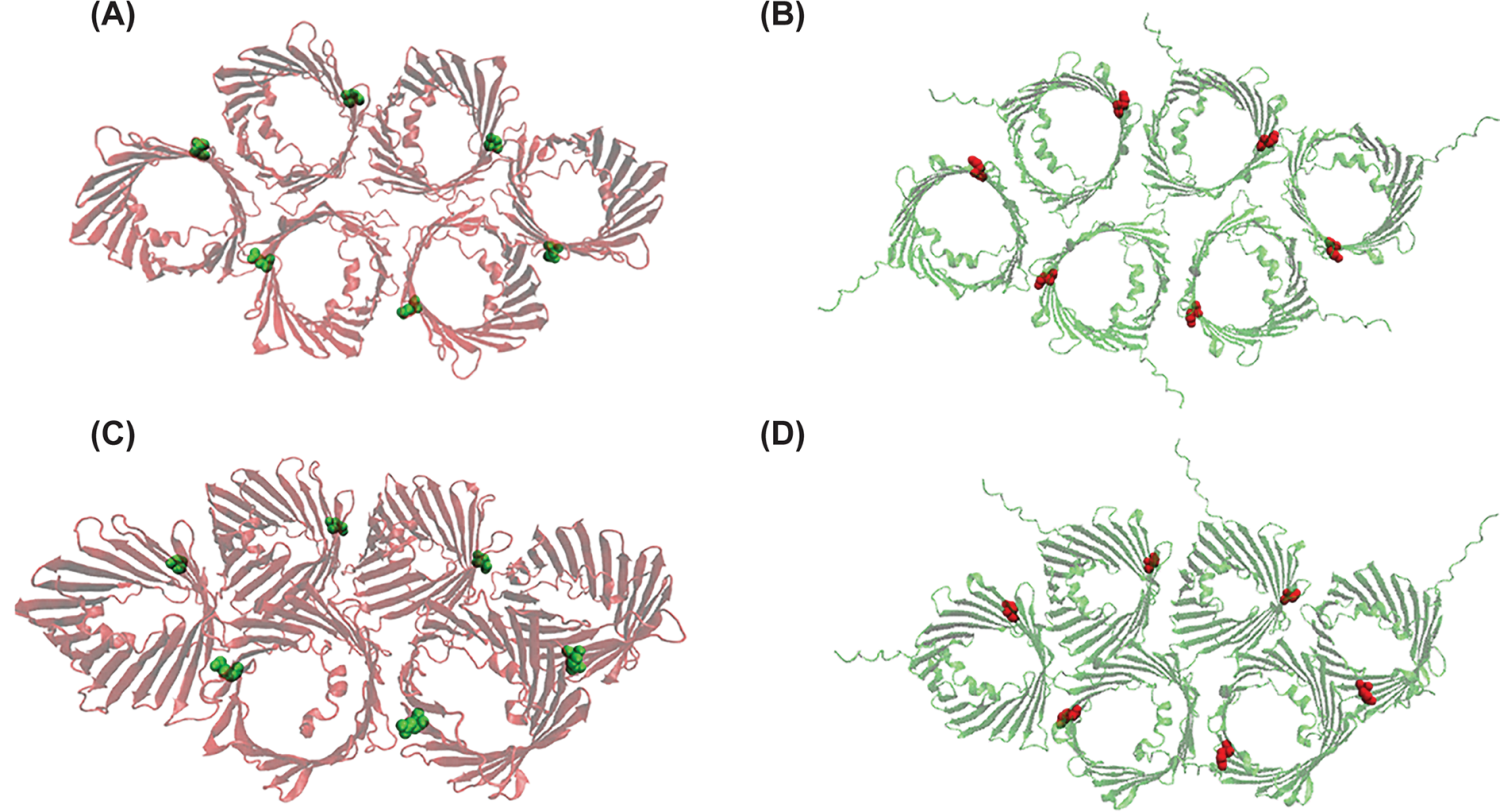
Comparison of the oligomeric structure of yeast Por1 with a modeled oligomer of human VDAC2 The hexameric structure of Por1 (PDB ID: 9JVQ) and the geometrically reconstructed hexamer of VDAC2 (modeled from AlphaFold P45880 and oligomerized via VMD software) are shown in red and green, respectively. (**A,B**) Top views of Por1 and VDAC2 oligomers; (**C,D**) side views inclined at an angle of 45°C. Residues 73 in Por1 and 84 in VDAC2 are highlighted using a VDW representation.

The idea of VDAC oligomerization relates to the pioneering works of Carmen Mannella and coworkers, who showed by electron microscopy such organization in the OMM of *N. crassa* [9]. This view was further supported by AF microscopy, which showed the sieve-like aspect of the surface of OMM containing assemblies of up to 20 pores [10]. Takeda et al. recently purified the homo-hexameric complex of yeast porin (Por1) in mitochondria from *Saccharomyces cerevisiae* [11]. Porin complex was purified in its native state under mild-detergent conditions from mitochondria, and the structure was solved via cryo-EM at a resolution of 3.2 Å, exhibiting a molecular weight of about 450–480 kDa according to the gel-filtration and Blue Native (BN) PAGE. The identified interfaces and residues, as schematized in Table 1, establish a network of hydrogen bonds between side chains and a series of electrostatic interactions that stabilize the structure into two mutually interacting trimeric forms. Notably, these residues exhibit a high degree of evolutionary conservation (as illustrated in Figure 1), and native human VDACs also show a similar molecular weight of 450–480 kDa in BN-PAGE [12–14], supporting the hypothesis that human VDAC isoforms may arrange in a similar manner as shown in Figure 2.

**Table 1:** Conservation scheme of residues mediating VDAC oligomerization, based on sequence alignment between yeast Por1, and human VDAC isoforms sequence

Residues	Localitazion	Conservation score	Chemical properties
T33	β1	*(11)	Polar
T35	β1	8	Polar
N37	Loop	9	Polar
Q194	β13	*(11)	Polar
D228	β15	*(11)	Polar with negative charge
S231	β16	7	Polar
Q249	β17	*(11)	Polar
R252	Loop	9	Polar with positive charge

The table reports the conservation scores for each residue participating in the formation of the VDAC hexamer oligomer related to yeast Por1 sequence. Scores were determined using the Jalview software, employing the Livingston & Barton algorithm [15]. A score of * (11) indicates absolute identity, while + (10) represents total conservation of physicochemical properties. Scores from 9 to 1 indicate decreasing levels of conservation, with 9: very high and 8: high. 0 denotes no conservation.

Zalk et al. [16] used cross-linking and immunoblotting techniques on mitochondria isolated from mouse liver, showing VDAC1 dimers (∼69 kDa), trimers (∼95 kDa), and tetramers (∼137 kDa). Reports available so far indicated that the formation of these oligomeric assemblies is promoted by VDAC up-regulation [17], mitochondrial stress and apoptosis stimuli [18], inflammation [19,20], low pH [21], and lipid composition of OMM [22,23].

A common dimeric assembly of VDAC, involving an interface between β-strands 1, 17, 18, and 19, has been reported by independent groups and is conserved across human VDAC1 [4], rat VDAC1 [24], and zebrafish VDAC2 [7]. However, low pH can regulate VDAC1 dimerization *in vitro*: under acidic conditions, mouse VDAC1 rearranges into a stable dimer with an interface between β-strands 2 and 5, where glutamate 73 (E73) becomes protonated and may form a hydrogen bond with S43 [21].

Daniliidis et al. demonstrated, using both cryo-EM and NMR spectroscopy, that VDAC1 dimer can interact with the anti-apoptotic protein B-cell lymphoma-extra large (BCL-xL), revealing a potential correlation between VDAC1 dimer and apoptosis activation [25].

In their study, they showed that the N-terminus can be displaced from the pore interior to interact with specific binding partners. VDAC1 undergoes oligomerization in the negatively charged detergent cholate and in negatively charged liposomes, but not in zwitterionic detergents CHAPS or LDAO. The extent of oligomerization strongly correlated with the degree of N-terminal helix exposure. The dimeric VDAC1 assembly was observed in nanodiscs, with the dimerization interface in the region of the β-barrel wall where the N-terminal helix is anchored, as it was also reported in [26]. This finding is in partial agreement with the proposed role of the N-terminal region, exposed by VDAC1 oligomers, as responsible for interactions with mt-DNA [19]. Additionally, magic-angle spinning NMR spectroscopy performed on human VDAC2 reconstituted into lipid bilayers [27] revealed high flexibility and mobility within the first 12 N-terminal residues, which may influence VDAC2’s interactions with metabolic partners [28] and potentially modulate its gating mechanism as suggested by Maurya et al. [29]. Figure 2 compares the six protomers oligomer described in [11] with a reconstruction of a similar oligomer of human VDAC2. To be noted are the locations of the residue 73 (yeast) or 84 (the homologous position in VDAC2), which stand almost at the interface between single protomers, and the protruding N-terminal sequence of VDAC2, whose structure is not predicted as ordered by AlphaFold.

VDAC1 oligomerization can also be promoted by lipid polarity and lipid composition of the OMM. This was proposed in early experiments by Mannella et al., who demonstrated the formation of the hexameric assembly upon partial delipidization of the *N. crassa* outer membrane [9]. Using VDAC1 reconstituted into giant unilamellar vesicles and fluorescence cross-correlation spectroscopy, Betanelli et al. showed that phosphatidylglycerol significantly enhances VDAC oligomerization in the membrane, whereas cardiolipin disrupts VDAC supramolecular assemblies [22].

When reconstituted into liposomes composed of phosphatidylcholine (PC), phosphatidylethanolamine (PE), and cholesterol at a physiologically relevant ratio (PC/PE/Chol ∼60/30/10), VDAC1 forms parallel-packed assemblies, as visualized by atomic force microscopy [23]. Notably, increasing cholesterol content promotes larger packed assemblies [23].

Far less is known about the oligomeric assembly of VDAC2 and VDAC3. Dimeric states of reconstituted human VDAC2 were observed in both LDAO micelles and large unilamellar vesicles [7,30]. Additionally, VDAC2 interacts with TOM complexes and might promote the formation of supercomplexes as recently documented by Callegari et al. [8]. VDAC2 can stabilize PTEN-induced kinase 1 (PINK1)—a protein associated with Parkinson’s disease and involved in mitophagy [31]. It was identified through cryo-EM at 3.1 Å that human VDAC2 dimers are mediated by disulfide bridges between residues C47 and C76, potentially conserved also in the rat ortholog [32]. While the three-dimensional structure of VDAC3 remains to be resolved, recent high-resolution mass spectrometry studies have mapped, for the first time, intra- and inter-molecular disulfide bonds in rat VDAC3 purified from mitochondria [33–35]. These linkages might enable VDAC3 to form dimers (homodimers or heterodimers with VDAC1/2) or higher-order oligomeric assembly [34]. Also, the potential of disulfide bridge formation between homo- or hetero-formed VDAC isoform oligomers should be a next point of investigation.

## Oligomeric VDAC: diverse roles in mitochondrial homeostasis and cell fate

Many reports have explored the connection between VDAC1 oligomerization and apoptosis [36,37]. This molecular mechanism was initially hypothesized by Shoshan-Barmatz et al. [38], correlating VDAC1 up-regulation with the apoptosis sensitivity, due to the oligomerization arrangement of VDAC1, which creates a pore large enough to allow the passage of apoptotic inducers, such as cytochrome c [39]. Hosaka and co-workers crystallized human VDAC1 dimers using bicelle method and predicted a heptameric structure mediated by hydrophobic interactions with β-strands, hydrophilic interactions with loop regions, and protein–lipid interactions [26]. However, the structure was not arranged to guarantee the passage of large molecules, but like an array, as reported in [9–11]. Recent structural work suggests that during oligomerization, the N-terminal α-helix—typically residing inside the pore—can be exposed [25]. This transition allows VDAC1 to inhibit anti-apoptotic BCL-2-like proteins (such as BCL-xL) and trigger cytochrome c release via pro-apoptotic proteins (e.g., BAK) [25]. This VDAC1 N-terminal exposure is also implicated in the interaction with mtDNA, between three positively charged residues (K12, R15, K20) in the N-termini and the negatively charged backbone of mtDNA. This interaction promotes VDAC1 oligomerization, the release of mtDNA itself, and triggers the activation of the cGAS-STING pathway in mammals [25]. In yeast, hexameric porin regulates mtDNA retention; however, the molecular mechanism does not involve the N-terminal positive charges; furthermore, the cooperation with nucleases in mitochondria or cytosol is of relevance [11].

In mammalian models, reduced mtDNA release—driven by the suppression of VDAC1 oligomerization—is also coupled with Parkin activity [40]. Specifically, Parkin-mediated ubiquitination of VDAC1 at lysine 53 (K53) prevents oligomerization, thereby inhibiting cGAS-STING-driven inflammation in hepatic stellate cells.

A novel mitochondria quality control distinct from mitophagy has been shown by Prashar and coworkers, in which damaged inner membranes are selectively removed. In this context, VDAC1 oligomers form large pores that allow herniation of IMM vesicles (VDIMs) to the cytosol [41]. These herniating inner mitochondrial membrane (IMM) structures are then engulfed by nearby lysosomes through a microautophagy-like process, aided by endosomal sorting complex required for transport machinery. VDIM formation increases under oxidative stress and requires calcium release through lysosomal transient receptor potential mucolipin 1 (TRPML1) channels, inducing VDAC1 oligomerization [41]. Since most mitochondrial proteins are nuclear-encoded, the organelle relies on sophisticated machinery to sort precursors. In 2019, Ellenrieder et al. and Sakaue et al. [42–44] identified VDAC (Porin) as a key regulator linking metabolism to mitochondrial biogenesis. VDAC modulates the TOM complex, the outer membrane’s primary entry gate, by facilitating the transition between its trimeric and dimeric forms. The dimeric form of the TOM complex lacking Tom22 is specifically required for the import of intermembrane space MIA pathway substrates. Additionally, VDAC interacts with carrier precursors in the intermembrane space, recruiting TIM22 complexes to ensure efficient transfer to the inner membrane translocase. The work by Takeda et al. [11] suggests that this role in protein import is maintained within VDAC hexameric assembly in yeast, though further studies are required to determine whether this function is conserved across all three mammalian isoforms (VDAC1–3).

VDACs are also players in mitochondria-endoplasmic reticulum (ER) contact sites (MERCs), known to regulate mitochondrial calcium transfer, but recently they were also proposed as phospholipid scramblases [30]. Both human VDAC1 and VDAC2 catalyze transbilayer phospholipid translocation through a molecular mechanism not related to their channel activity. VDAC dimers, with interfaces involving strands β1, 2, 4, 18, and 19 (specific residues are shown in Table 2), facilitate lipid flipping where polar residues induce membrane thinning and water defects. The recently attributed scramblase activity of VDACs conflicts with the proposed oligomeric assembly [11], since the dimeric interface indicated in [30] appears to be buried within the confined space of the hexamer. Therefore, either a dynamic assembly of VDAC protomers must be demonstrated, resulting in a range of intermediate forms (dimers and trimers), or the scramblase pathway must be redefined. In support of the former hypothesis, the authors demonstrated that VDAC2 has a stronger propensity to dimerise in membrane vesicles and exhibits faster lipid scrambling activity than VDAC1 *in vitro* [30]. This is a typical case of a feature that must be reconsidered in light of the oligomer structure. The residue E73 (or E84 in VDAC2) is central to both the polar pathway supporting lipid scrambling activity and hexokinase binding. However, it is less available for interaction due to its location at the protomer interface (see also Figure 2). The presence of VDAC, however, seems necessary for scramblase activity since genetic studies in yeast underscore the significance of this role: mitochondria lacking Porin exhibit an order of magnitude reduction in phospholipid import rates [30]. Furthermore, hexameric porin can functionally act as scramblase [11]. This evolutionary conservation from yeast porin to mammalian VDACs highlights its fundamental importance in mitochondrial lipid homeostasis (for a detailed review [45]).

**Table 2:** Amino acid residues involved in VDAC1 and VDAC2 scrambling activity and their polarity features

Residues	Localitazion	Polarity
T33 (T44)	β1	Moderate
S35 (S36)	β1	Moderate
S43 (S44)	β2	Moderate
E73 (E84)	β4	Very High (negative charge)
T77 (T88)	β4	Moderate
Y247 (Y258)	β17	Low
Q249 (Q260)	β17	Elevate

The table lists the primary residues participating in lipid flipping (residues of VDAC2 in brackets), as identified by Jahn et al. and supported by CGMD simulation following valine substitution (except for E73) [30]. Polarity is defined based on physicochemical properties, with hydrophilicity values derived from the Kyte-Doolittle hydropathy scale [46].

## Interacting partners extend VDAC functions: inter-organelle relationships, apoptosis, and quality control

The strategic positioning of VDACs has revealed their pivotal role in inter-organellar communication. As already mentioned, phospholipid scrambling activity of VDACs was observed. This is necessary to transport phosphatidic acid (PA) from the ER to mitochondria for cardiolipin synthesis and phosphatidylserine (PS) for PE synthesis at the matrix side of the IMM. In budding yeast, Por1 interacts with the IMM proteins Mdm31, which is required for CL accumulation, and Mdm35, which interacts with Ups1 and Ups2 for PA and PS transport. Depletion of porin decreases CL levels and destabilizes Ups1 and Ups2. Moreover, down-regulating VDAC1/2 in HeLa cells affects CL accumulation, highlighting the conserved role of VDAC in lipid homeostasis in yeast and mammals [47].

At MERCs, VDAC1 participates in multi-protein complexes with the ER-localized inositol 1,4,5-trisphosphate receptor (IP3R) and the chaperone glucose-regulated protein 75 (GRP75) [48]. This IP3R-GRP75-VDAC1 complex mediates efficient calcium (Ca^2+^) transfer from the ER to the mitochondrial intermembrane space, thereby regulating calcium homeostasis and bioenergetics [49]. Interestingly, recent evidence indicates that VDAC2 also interacts with IP3R [50]. IP3R and VDAC2 were found to form a complex with the lamin B receptor (LBR) during mitosis. This IP3R-LBR-VDAC2 complex regulates mitochondrial calcium influx from ER to sustain the energy production required in the metaphase-anaphase transition [51]. At MERCs, VDACs also interact with the insertase ER membrane protein complex (EMC) [52]; however, the functional role of the VDAC-EMC complex has yet to be clarified.

Mitochondrial calcium homeostasis is also regulated by mitochondria-lysosome associations; specifically, the lysosomal channel TRPML1 binds preferentially to VDAC1, tethering the organelles to mediate calcium transfer from lysosome to mitochondria [53]. Furthermore, VDAC2 tethers mitochondria to endosomes by binding the phosphoinositide 3-kinase, promoting endosome maturation [54].

Cellular metabolism is also regulated by the interaction of cytosolic hexokinases I and II (HKI/HKII) with VDACs, facilitating preferential access of mitochondrial ATP to glycolysis and supporting anabolic metabolism in highly proliferative cells. This interaction confers resistance to apoptosis by limiting cytochrome c release and inhibiting mitochondrial outer membrane permeabilization, thereby linking metabolic rewiring to cell survival in cancer. Recent evidence indicates that disruption of the HKII-VDAC1 complex induces mitochondrial calcium influx and stress responses that drive VDAC oligomerization, thereby promoting activation of the NLRP3 inflammasome [55]. *In vitro*, HKs partially reduce VDAC1 channel conductance in an artificial membrane [56]. While VDAC1 is the dominant binding partner for HKs, evidence suggests that VDAC2 may also support hexokinase association in certain cellular contexts [57]. The model of the interaction and binding of HK-VDAC should now be redesigned following the elucidation of the hexameric oligomer. In the oligomer Q73, the yeast residue that corresponds to E73—the amino acid that has been identified as being involved in hexokinase binding—is indeed spatially restricted at the interfaces between the protomers, as can be seen in Figure 2. This structural position restricts the possibility of interaction between the N-terminus of HK and the barrel. This raises new questions about the mechanism of triggering interaction between VDAC and HK, as well as pore closure and apoptosis interference. Thinning of the membrane at the Q73/E73 position [57] has been shown to be an important feature of the mechanism, since it favors insertion of the HK N-terminus into the membrane along the Q73/E73-containing beta strand. Following the results in [57], membrane thinning in the oligomeric model should occur between single protomers and not just between a monomer and the surrounding phospholipid layer. If this phenomenon were to be repeated among the plethora of protomer interfaces, it could strongly destabilize the membrane. Overall, this event should be reconsidered and reinterpreted. In general, we would like to suggest that, from now on, models of interaction between HK and VDAC should take into consideration that VDAC is organized as an oligomer in the membrane. The molecular docking of HK upon VDAC should be demonstrated using the oligomer. Also, the role of E73, which we consider important, should be explained in light of the oligomeric organization.

VDACs also have a role in mitochondrial quality control, either as ubiquitinated substrates or as interacting proteins. While VDAC1 and VDAC3—but not VDAC2—are targets for Parkin-mediated ubiquitination during mitophagy [58,59], all isoforms appear to interact with Parkin and regulate its mitochondrial recruitment [58,59]. Recently, Callegari et al. utilized cryo-EM to resolve a complex structure featuring a dimeric human PINK1 situated above TOM-VDAC complexes. In this arrangement, a central VDAC2 dimer stabilizes PINK1 [8]. Additionally, VDAC1 interacts with the 18-kDa translocator protein, which can promote excessive reactive oxygen species (ROS) production, potentially counteracting Parkin-mediated ubiquitination [60]. VDAC1 was found to interact with prohibitin 2 (PHB2), an IMM protein that is exposed during stress-induced mitophagy [61]. Specifically, following mitochondrial depolarization, VDAC is essential for PHB2 exposure and the subsequent PHB2-ATG8 interaction, as observed in *Drosophila* models [61].

Members of the BCL-2 family of apoptosis regulators further exemplify isoform-specific modulation of VDAC. Pro-apoptotic proteins B-cell lymphoma 2 antagonist/killer (BAK) and B-cell lymphoma 2-associated X protein (BAX) interact with VDAC1 to promote oligomerization, whereas anti-apoptotic proteins such as BCL-2, BCL-xL, and MCL-1 bind VDAC to suppress conductance changes and inhibit apoptosis [62]. VDAC2 exerts dual role in cell death: it is required for BAX-mediated apoptosis, but conversely inhibits BAK-mediated apoptosis (as extensively reviewed in [63,64]. *VDAC2* genetic ablation disrupts BAK sequestration and BAX recruitment, underscoring a functional specialization of VDAC2 distinct from VDAC1 [14,64–66]. VDAC3, although less abundant, has also been implicated in apoptotic regulation. In particular, BCL-xL and MCL-1 are also found to be associated with it. The VDAC1/3-BCL-xL interaction facilitates Ca^2+^ transport across the MOM and Ca^2+^ matrix accumulation, thus increasing mitochondrial permeability and promoting apoptosis [67,68].

Additional endogenous interactors, several misfolded proteins related to neurodegenerative diseases [69–74], and components of the cytoskeleton [75–79] further diversify the VDAC interactome and contribute to isoform-dependent regulation of mitochondrial calcium handling, ROS production, and bioenergetics [80]. Moreover, VDACs are essential for male fertility, regulating sperm mitochondrial integrity, motility, and redox balance; their role in male reproductive physiology and their interactions have been comprehensively discussed in [81].

## Pharmacological modulation of VDAC: targeting channel activity, oligomeric assembly, and interactome

Beyond endogenous proteins, a growing number of small molecules with therapeutic relevance have been identified that interact with VDACs and modulate their structure and function. Several VDAC-targeting compounds have already been extensively reviewed in [82]. A new paradigm emerging today from the elucidation of oligomerization structural details is the ability to design interacting molecules by chemical selection and design based on informatic tools. Additionally, the discovery of natural or synthetic molecules recognized as VDAC effectors on the basis of functional results could now be reinterpreted in light of the structure. The domains of interaction between single protomers also appear to be relevant now. A mechanism for inhibiting oligomer formation could indeed come from molecules interacting outside the barrel, at the interface between protomers, or between protomers and phospholipids or cholesterol. This new approach will enhance in the near future the design of further molecules, sometimes based on the known interactors.

In this light, we specifically focus only on recent or less frequently reported studies showing the effects of small molecules.

More recent evidence identifies the most potent erastin-like compound, the quinazolinone derivative X1 (5-chloro-N-[4-chloro-3-(trifluoromethyl)phenyl]-2-(ethylsulfonyl)-4-pyrimidinecarboxamide), as a small-molecule modulator of VDAC1, identified through structure-guided screening approaches, that selectively induces metabolic stress in highly oxidative and glycolytic tumor models [83]. In addition, some natural compounds are capable of directly altering the function of VDAC channels. Celastrol, a bioactive triterpenoid extracted from *Tripterygium wilfordii*, covalently modifies cysteine residues on VDAC2, thereby disrupting VDAC2-mediated mitochondrial permeability transition and triggering ROS-dependent apoptosis and ferroptosis in cancer cells [84]. Similarly, lycorine induces VDAC2-dependent mitochondrial dysfunction characterized by aberrant mitochondrial morphology, increased membrane potential, disrupted ion homeostasis, and enhanced oxidative stress, ultimately inhibiting multiple myeloma growth, likely through perturbation of the VDAC2-HK II interaction [85].

Recent work has identified VDAC-targeting small molecules, among which is VBIT-4 (voltage-dependent anion channel binding inhibitor-4); this is a small-molecule inhibitor designed to specifically target VDAC1 oligomerization. By preventing VDAC1 self-association, VBIT-4 inhibits the formation of large-conductance pores that facilitate the release of apoptogenic factors such as cytochrome c. Consequently, VBIT-4 has emerged as a promising pharmacological tool for modulating mitochondria-mediated apoptosis and mitigating cell death in models of neurodegenerative and inflammatory diseases [19,86–90]. Although VBIT-4 has been primarily characterized for VDAC1, partial conservation of its proposed binding site in VDAC2 and VDAC3 suggests potential cross-isoform activity. However, recent evidence indicates that VBIT-4 predominantly acts as a membrane-active compound that perturbs lipid bilayer integrity and increases permeability in a largely VDAC-independent manner, leading to cytotoxic effects at micromolar concentrations and raising concerns regarding its mechanistic specificity [91]. Conversely, SW016789 acts as a VDAC activator, enhancing calcium flux and insulin secretion in pancreatic β-cells, underscoring the therapeutic potential of VDAC modulation beyond oncology and suggesting that isoform-specific expression patterns may dictate tissue-selective outcomes [92].

A particularly significant advance in the field is the identification of molecules discovered through an integrated *in silico* and experimental screening strategy targeting a regulatory pocket on VDAC1 that overlaps with the NADH-binding site. Maldonado and co-workers identified a small molecule, SC-18, which induces mitochondrial dysfunction characterized by reduced membrane potential, impaired ATP production, and altered oxygen consumption in hepatocarcinoma cells. Given the conservation of this regulatory pocket, SC-18 is predicted to interact with all three VDAC isoforms: this is consistent with observations that its effects persist in cells lacking individual VDAC isoforms, supporting a model of functional redundancy and cooperative regulation among VDAC family members [93,94]. In 2024, Lolicato group identified small molecules, known as VA (for VDAC1 antagonist) compounds, that similarly target a conserved regulatory region associated with NADH in VDAC1. These molecules alter channel voltage gating, selectivity, and metabolite transport, and they exhibit potent cytotoxicity against highly glycolytic breast cancer cells and patient-derived organoids from cholangiocarcinoma [95,96]. Furthermore, WEHI-3773 was identified through a cell-based phenotypic screening approach aimed at discovering small molecules capable of modulating VDAC2-dependent apoptotic signaling. Rather than acting as a simple pore blocker, WEHI-3773 binds to VDAC2 and differentially regulates its interaction with pro-apoptotic BCL-2 family proteins, inhibiting BAX mitochondrial localization while enhancing BAK-mediated apoptosis [97].

Additional evidence for pharmacological targeting of VDAC has emerged from studies identifying small molecules that modulate VDAC-dependent calcium signaling. Efsevin, an antiarrhythmic compound, binds selectively to VDAC2 and enhances mitochondrial Ca^2+^ uptake, thereby influencing metabolic activity and excitation-contraction coupling, and providing direct proof that individual VDAC isoforms can be selectively targeted by small molecules to modulate calcium flux independently of metabolite transport [98]. More recently, a novel VDAC-binding small molecule, known as NCATS-SM0225, identified through high-content screening, was shown to perturb ER-mitochondria calcium homeostasis and induce cancer cell death via activation of the PERK-STIM1 signaling axis, highlighting a non-canonical mechanism by which VDAC ligands can influence cellular fate through regulation of inter-organelle communication and calcium signaling rather than direct pore blockade or opening [99].

Peptide-based modulators targeting VDAC protein–protein interactions have emerged as a complementary therapeutic strategy. Peptides derived from VDAC1 sequences or from interaction motifs of BCL-2 family proteins act as decoys that disrupt anti-apoptotic complexes at the mitochondrial surface, promoting apoptosis in cancer cells [100,101]. The mitochondrial-targeted NHK1 peptide, designed by the Messina group [73], copies the N-terminal domain of HK1 and prevents the binding of VDAC1 to misfolded proteins, including SOD1 mutants and Aβ oligomers. NHK1 enhanced respiratory function and restored ATP-associated respiration in ALS and AD models [72,73,102]. While research has primarily focused on VDAC1, similar interfaces in VDAC2 and VDAC3 suggest that isoform-specific peptides could be engineered to precisely regulate redox signaling and mitochondrial quality control. As summarized in Table 3, several small molecules and peptides modulate VDACs, with distinct target isoforms, mechanisms of action, and biological effects.

**Table 3:** Summary of VDAC-targeting small molecules and peptide-based modulators

Isoform target	Compound	Mechanism of action	Biological effect	Ref.
VDAC1	X1 (quinazolinone derivative)	Erastin-like modulator; induces metabolic stress	Selective metabolic stress in oxidative and glycolytic tumor models	[83]
	VBIT-4	Inhibits VDAC1 oligomerization	Anti-apoptotic in neurodegenerative/inflammatory models	[19,86–90]
	VDAC antagonist (VA) compounds	Alters voltage gating, selectivity, metabolite transport	Cytotoxic in glycolytic breast cancer and cholangiocarcinoma organoids	[95,96]
	SW016789	Activates VDAC; enhances Ca^2+^ flux	Increases insulin secretion in pancreatic β-cells	[92]
	NHK1 peptide	Prevents VDAC1 interaction with misfolded proteins (SOD1, Aβ)	Restores mitochondrial respiration in ALS and AD models	[72,73,102]
	VDAC1-derived peptides / BCL-2 interaction motif peptides	VDAC1-derived peptides/BCL-2 interaction motif peptides	VDAC1-derived peptides/BCL-2 interaction motif peptides	[100,101]
	NCATS-SM0225	Disrupts ER-mitochondria Ca^2+^ homeostasis; activates PERK-STIM1 axis	Selective cancer cell death via calcium dysregulation	[99]
VDAC2	Celastrol	Covalent modification of cysteine residues; disrupts mitochondrial permeability transition	ROS-dependent apoptosis and ferroptosis in cancer cells	[84]
	Lycorine	Induces mitochondrial dysfunction; perturbs VDAC2-HK II interaction	Inhibits multiple myeloma growth	[85]
	WEHI-3773	Modulates VDAC2-BCL-2 interactions; inhibits BAX recruitment and enhances BAK activity	Differential regulation of apoptosis in cancer cell	[97]
	Efsevin	Enhances mitochondrial Ca^2+^ uptake	Modulates metabolism and excitation-contraction coupling	[98]
VDAC1/2/3	SC-18	Reduces membrane potential, ATP production, and oxygen consumption	Mitochondrial dysfunction in hepatocarcinoma cells	[93,94]

The table lists compounds and peptides reported to interact with VDACs, their target isoforms, identification strategies, mechanisms of action, and primary biological effects.

## Conclusions

In conclusion, the recent molecular elucidation of the yeast Por1 oligomeric structure opens vast new avenues for VDAC research, although conflicting evidence still exists regarding the oligomeric arrangement of VDAC, specifically whether it assembles into ordered arrays or forms large-scale pores that allow molecules to escape from mitochondria. In the light of these results, which are likely translatable to other species, much data and information obtained in previous years should probably be reconsidered. It is important to note that oligomerization has now moved beyond speculation and models to be given a precise definition. This development is reminiscent of the moment in 2008 when the true structure of VDAC1 was published, which differed from the structures of bacterial porins and other hypotheses. Consequently, the mechanisms by which partner proteins bind to such hubs—and the proposed function of these oligomers as “mega-pores”—should be re-evaluated in the context of this finding, which represents the most significant breakthrough in the field in a decade. The hypothesis of the formation of hetero-oligomers between different mammalian isoforms should also be considered at this stage, since the residues responsible for the interactions between the protomers are conserved. The hub in the mitochondrial surface is now beginning to unveil its mechanisms.

## Perspectives

- Highlight the importance of the field: VDAC is increasingly considered a hub for the organization of bioenergetic metabolism and its intersection with cellular structures. Its role in mitochondria maintenance and dysfunction is considered a switch for pharmacological actions.
- Provide a summary of the current thinking: The structural definition of its oligomeric organization is the main recent breakthrough and opens enormous new perspectives for knowledge.
- Comment on future directions: The detailed mechanisms of action of drugs and interacting partners can now be studied in relation to the oligomer structure. This means that previously unclear or inconclusive results can now be correctly evaluated. The design of molecules targeting VDAC for promising new therapeutic approaches will be enhanced.

## Open Access

Open access for this article is provided via our Subscribe to Open programme, made possible through the support of our subscribing institutions. Learn more here: https://portlandpress.com/pages/open_access_options_and_prices#conditional

## Abbreviations

BAK: B-cell lymphoma 2 antagonist/killer
BAX: B-cell lymphoma 2-associated X protein
BCL-xL: B-cell lymphoma-extra large
BN: Blue Native
cryo-EM: cryo-electron microscopy
ER: endoplasmic reticulum
EMC: ER membrane protein complex
GRP75: glucose-regulated protein 75
HKI/HKII: hexokinases I and II
IMM: inner mitochondrial membrane
LBR: lamin B receptor
MERCs: mitochondria-endoplasmic reticulum contact sites
mtDNA: mitochondrial DNA
MSA: Multiple sequence alignment
OMM: outer mitochondrial membrane
PA: phosphatidic acid
PC: phosphatidylcholine
PE: phosphatidylethanolamine
PS: phosphatidylserine
PHB2: prohibitin 2
PINK1: PTEN-induced kinase 1
ROS: reactive oxygen species
TRPML1: transient receptor potential mucolipin 1
VBIT-4: voltage-dependent anion channel binding inhibitor-4
VDAC: voltage-dependent anion-selective channel
